# Chill out: physiological responses to winter ice-angling in two temperate freshwater fishes

**DOI:** 10.1093/conphys/cox027

**Published:** 2017-04-27

**Authors:** Michael J. Louison, Caleb T. Hasler, Graham D. Raby, Cory D. Suski, Jeffrey A. Stein

**Affiliations:** 1 Illinois Natural History Survey, 1816 South Oak Street, Champaign, IL61820, USA; 2 Department of Natural Resources and Environmental Sciences, University of Illinois at Urbana-Champaign, 1102 South Goodwin Avenue, Urbana, IL61801, USA; 3 Great Lakes Institute for Environmental Research, University of Windsor, 2601 Union Street, Windsor, Ontario, Canada N9B 3P4

**Keywords:** Catch-and-release, cortisol, ice fishing, lactate, RAMP, stress response

## Abstract

A large body of research has documented the stress response of fish following angling capture. Nearly all of these studies have taken place during the open-water season, with almost no work focused on the effects of capture in the winter via ice angling. We therefore conducted a study to examine physiological disturbance and reflex impairment following capture by ice-angling in two commonly targeted species, bluegill *Lepomis macrochirus* and yellow perch *Perca flavescens*. Fish were captured from a lake in eastern Wisconsin (USA) and sampled either immediately or after being held in tanks for 0.5, 2 or 4 h. Sampling involved the assessment of reflex action mortality predictors (RAMP) and a blood biopsy that was used to measure concentrations of plasma cortisol and lactate. The capture-induced increase in plasma cortisol concentration was delayed relative to responses documented in previous experiments conducted in the summer and reached a relative high point at 4 h post-capture. Reflex impairment was highest at the first post-capture time point (0.5 h) and declined with each successive sampling (2 and 4 h) during recovery. Bluegill showed a higher magnitude stress response than yellow perch in terms of plasma cortisol and RAMP scores, but not when comparing plasma lactate. Overall, these data show that ice-angling induces a comparatively mild stress response relative to that found in previous studies of angled fish. While recovery of plasma stress indicators does not occur within 4 h, declining RAMP scores demonstrate that ice-angled bluegill and yellow perch do recover vitality following capture.

## Teaser

We quantified physiological disturbance and reflex impairment in ice-angled bluegill and yellow perch. Rises in plasma cortisol and lactate following capture were lower than those found in prior studies done in summer. Recovery of plasma metrics did not occur within 4 h of capture, however, reflex responsiveness levels did recover.

## Introduction

The ability of an organism to successfully respond to stress can have ramifications for fitness, and the extent of physiological disturbance accompanying a given stressful situation may vary among individuals or species (Koolhaas et al., 1999; Pottinger, 2010). The response of organisms to stress may also be influenced by environmental conditions, including temperature (Wieser et al., 1986; Lankford et al., 2003; Davis, 2004). In the case of fish, studies have indicated that high temperatures can lead to a more pronounced hormonal (Barton and Schreck, 1987; Jaxion-Harm and Ladich, 2014) or metabolic response (Kieffer et al., 1994; Sfakianakis and Kentouri, 2010). Cold temperatures, on the other hand, have often been found to dampen the magnitude of this response (Van Ham et al., 2003; Davis, 2004; Guderley, 2004).

One area in which the effects of stress on fish has been heavily studied is the physiological response of fish to recreational or commercial capture. This includes capture via netting (Donaldson et al., 2011) or hook-and-line angling (Arlinghaus et al., 2009; Brownscombe et al., 2015). In the case of angling, the widespread practice of catch-and-release has led to a research focus on post-release stress and mortality (Cooke and Suski, 2005; Cooke and Schramm, 2007). A host of studies have demonstrated that angling capture and handling may lead to significant physiological disturbance (Cooke et al., 2002; Cooke and Suski, 2005; Meka and McCormick, 2005; O’Toole et al., 2010) as well as mortality (Dubois et al., 1994; Davis, 2007; Gutowsky et al., 2015) following release. The degree of stress experienced by a captured fish may be influenced by biotic (Cooke and Suski, 2005; Clark et al., 2012) and abiotic (Meka and McCormick, 2005; Gingerich et al., 2007) factors, as well as how the fish is handled by the angler (Brydges et al., 2009; Cook et al., 2015). Generally speaking, high water temperatures have been associated with both greater physiological disturbance and a greater likelihood of mortality (Meka and McCormick, 2005; Gingerich et al., 2007; Gale et al., 2013). While the impacts of high temperatures on fish following capture via angling have indeed been reasonably well studied, the impacts of capture at low temperatures (for instance, in winter) have received less attention.

The lack of research on the response of fish to winter capture can be attributed to a variety of factors, including the uncomfortable working environment at low temperatures, the difficulty of capturing fish and issues in keeping equipment functional (Lavery, 2016). As a result, there is a paucity of studies on the physiological responses of freshwater fish to catch-and-release in cold winter conditions (but see Louison et al., 2017), save for examinations of post-release mortality (Dubois et al., 1994; Persons and Hirsch, 1994). This is a significant gap in our knowledge of how fish respond to angling stress, considering that ice-angling is a popular activity at higher latitudes (Deroba et al., 2007) and large numbers of captured fish are often released (~50% in some cases) (Margenau et al., 2003). Understanding the stress imposed by ice-angling on fish is important to fisheries managers who recommend to anglers best practices for handling captured fish (Cooke and Cowx, 2004; Cowx et al., 2010).

Earlier work has documented the stress response of northern pike *Esox lucius* to winter capture (Louison et al., 2017), however, additional work is needed to further our knowledge of how fish respond to the stress of ice-angling. One aspect that has not been examined is how ice-angling impacts the vitality of fish. A relatively new and relevant tool to assess this is the assessment of reflex responsiveness, through the use of reflex action mortality predictors (RAMP) (Davis, 2007, 2010). Using RAMP to quantify vitality (alternatively, reflex impairment) in fish has several advantages relative to laboratory-based measurements (for instance, levels of cortisol or lactate in the plasma), including the speed at which assessments can be performed and the lack of laboratory expertise necessary to perform them (Raby et al., 2012). RAMP has been used successfully to assess mortality risk in captured fish, but only during the open-water season (Raby et al., 2012; McArley and Herbert, 2014; Bower et al., 2016). By using both an assessment of plasma metrics and reflex responsiveness concurrently, we hope to provide a more comprehensive assessment of the response of bluegill *Lepomis macrochirus* and yellow perch *Perca flavescens* to winter capture.

To further our knowledge of the response of fish to winter capture, we conducted a study that examined bluegill and yellow perch following capture through the ice. These two species were selected because they are among the most commonly targeted by ice anglers throughout much of central and northeastern North America (Gaeta et al., 2013). While no data exists on release rates in winter for these species, summer release rates have been found to range from 67 to 99% (Gaeta et al., 2013). The objectives of this study were threefold: (i) to define how plasma stress metrics (cortisol and lactate) in bluegill and yellow perch respond to the stress of capture in the winter, (ii) to quantify reflex impairment and recovery of reflex function of these two species following ice angling capture and (iii) to characterize the concordance between reflex impairment and blood constituents assessed under winter conditions. Results from this study serve to fill a notable gap in the catch-and-release literature, and provide recommendations for what assessments may be most useful in describing the physiological status of winter captured fish.

## Methods

### Study site

**Figure 1:**
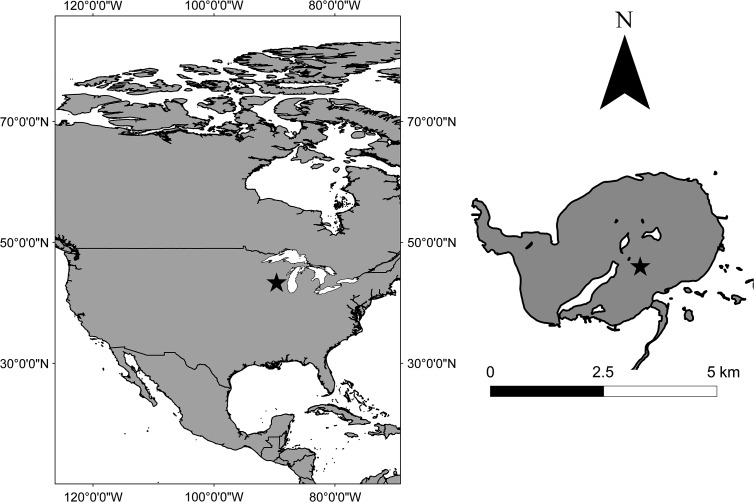
Study location, Fox Lake, Wisconsin, USA. The location of Fox Lake within North America, and the location of sampling within the lake are indicated by stars.

All sample collection took place on February 20 and 21, 2016 between 9:00 and 16:30 at Fox Lake (Fig. 1), a 1097 ha lake with a mean depth of ~2 m located in Dodge County, WI, USA (43.584845 N, 88.923569 W). Over the course of the 2 days, air temperatures fluctuated between 1 and 6°C (as measured with a hand-held thermometer), and water temperatures were recorded between 3.4 and 4.2°C. In addition to bluegill and yellow perch, heavily targeted sportfish in Fox Lake include muskellunge *Esox masquinongy*, largemouth bass *Micropterus salmoides*, northern pike *Esox lucius* and walleye *Sander vitreus* (Fox Lake Profile, Wisconsin Department of Natural Resources Website, http://dnr.wi.gov/lakes/lakepages/LakeDetail.aspx?wbic=835800).

### Fish capture and holding

All fish in this study were captured via ‘jigging’, a typical approach used by anglers to capture yellow perch and bluegill through winter ice. The fishing gear consisted of small (0.8 m), light-action jigging rods spooled with 1.8 kg monofilament line rigged with a size 2 jig (Barbed J-hook with a colourful, weighted head). Jigs were baited with either a live waxworm (*Galleria* spp.) or a small soft plastic lure, and slowly bobbed up and down about 0.5 m from the bottom by the angler at a depth of 1.5 m. Once a strike was felt, the angler quickly raised their fishing rod to lift the fish from the water, with the time from hooking to landing the fish never exceeding 5 s. Six experienced anglers were responsible for capturing the fish used in this study.

Upon capture, fish were quickly unhooked and either treated as a baseline sample (i.e. immediately sampled) or placed into one of several 68 l opaque plastic holding tanks containing fresh lake water. Water temperatures in the tanks were stabilized by ambient conditions and periodic water exchanges to hold between 3 and 5°C, and temperature was checked during times when no fish were being held using a dissolved oxygen and temperature probe (YSI^®^, Yellow Springs, OH, USA). Dissolved oxygen concentrations were never below 90% saturation at any point during the study. The duration of air exposure, from the time a fish was landed until it was transferred to holding tanks or sampling, was standardized at 60 s. This short handling period is realistic for ice-angled fish, which often are handled for less time than summer-angled fish (Dextrase and Ball, 1991). Additionally, unlike with larger species, ice-anglers that capture bluegill and yellow perch typically do not pause to take photos or otherwise show off their catch if it is to be released (Louison, personal observation). Fish were not included in the study if they were bleeding or if hooking took place deep in the throat or gills, potentially leading to injury that could confound the results (Cooke et al., 2011; Stein et al., 2012), however, fewer than five fish fell into this category during sampling. Fish randomly chosen as baseline samples were immediately sampled for blood as described below, with no RAMP testing taking place. RAMP scores were not assessed in the baseline group to avoid the introduction of bias resulting from each individual angler assessing RAMP immediately upon capture, and to ensure blood samples were drawn as rapidly as possible. Non-baseline fish were held in tanks for a period of 0.5, 2 or 4 h before being assessed for RAMP and having blood drawn.

### RAMP assessment, blood biopsy and plasma analysis

Each non-baseline fish was immediately tested for RAMP following the conclusion of its holding time (0.5, 2 or 4 h). Assessment of RAMP followed previously established protocols (Davis, 2007; Raby et al., 2012) and included the assessment of four metrics: ‘tail grab’ (whether or not the fish attempted to burst away in the holding tanks when grabbed on the caudal peduncle by the handler), ‘orientation’ (whether or not the fish righted itself within 3 s after being placed in its holding tank upside down), ‘body flex’ (whether the fish attempted to escape while being held out of the water around the midsection of its body) and ‘vestibular-ocular response’ (VOR, whether or not the fish rolled its eye to maintain contact with the handler after being rotated out of the water from normal orientation onto its side). Head complex (whether the fish continued to open its jaws and operculum in a normal ventilation pattern out of the water) was not included in our analyses because every fish that was captured showed impairment for this reflex. All RAMP assessments were performed by a single observer (GDR), with scores for each reflex either recorded as a 0 (present, no impairment), or a 1 (impaired/absent). If the response of a fish for any of the metrics was ambiguous, it was recorded as impaired. Overall RAMP score for each individual was taken as the proportion of the four reflexes that were impaired (higher scores = lower vitality).

Following completion of the assessment (which took 10–15 s) each fish was transferred to a foam sampling trough where 0.1–0.25 ml of blood was drawn *via* caudal puncture using a 1 ml heparinized syringe equipped with a 23 gauge needle. Blood was immediately centrifuged at 6000 RPM for 120 s to extract plasma, which was immediately stored in liquid nitrogen for transport back to the laboratory where it was subsequently stored at −80°C. Cortisol concentration in plasma was quantified using a commercially available ELISA Immunoassay kit (Enzo Life Sciences, Farmingdale, NY, USA), previously validated for use in fishes (Sink et al., 2008). Plasma lactate concentrations were quantified calorimetrically from perchloric acid extracts on a 96-well spectrophotometry plate based on methodology in Lowry and Passonneau (1972).

A total of 66 bluegill and 39 yellow perch were captured over the 2 days of ice-angling. Blood samples were obtained from a minimum of eight fish for each combination of species and holding time (Table 1) but insufficient plasma was extracted to conduct assays for both cortisol and lactate for some fish. In those cases, performance of only one of the two assays resulted in a reduced sample size for the other metric for that species × holding time combination (Table 1).

**Table 1:** Summary of sample sizes of bluegill and yellow perch for each sampling time point and analysis. In some cases, insufficient plasma resulted in the inability to run assays for both lactate and cortisol. The final number of individuals (*N*) for each treatment × time group for each metric is shown

	Holding time	*N* captured	Mean length (cm ± S.E.M.)	RAMP *N*	Cortisol *N*	Lactate *N*
Bluegill	Baseline	18	17.14 (±0.50)	NA	17	16
	30 min	18	16.52 (±0.38)	18	18	18
	2 h	17	17.93 (±0.52)	17	16	16
	4 h	13	16.17 (±0.69)	13	13	13
Yellow perch	Baseline	11	14.59 (±0.30)	NA	9	11
	30 min	11	16.45 (±0.69)	11	8	10
	2 h	9	15.06 (±0.73)	9	8	8
	4 h	8	16.30 (±1.02)	8	8	8

### Statistical analysis

To test whether plasma metrics differed across species or holding times (including baseline samples in the case of cortisol and lactate), we ran separate two-way analyses of variance (ANOVA) for cortisol and lactate. Each analysis included species, holding time and their interaction as fixed factors. Fish length was initially included as a covariate in both models, however, it was removed when it did not approach significance (Engqvist, 2005). In each case, homogeneity of variance was assessed using a Levene’s test, and normality was assessed via visual inspection of q–q plots. In the case of a significant main effect of holding time, pairwise differences were assessed using Tukey’s Honest Significant Difference (HSD) test. In the event of a statistically significant interaction term, pairwise differences were tested among species × holding time groups and main effects were ignored.

Binary logistic regression models were used to determine the effects of species, holding time and fish length on whether or not a fish showed any sign of reflex impairment. Holding time was treated as a categorical variable, and impairment (whether or not a fish had a non-zero RAMP score) was treated as the response variable. To assess whether reflex impairment was reflected by levels of plasma stress metrics (regardless of holding time), we ran an ordinal regression (Winship and Mare, 1984) for bluegill with RAMP score as the dependent variable, and cortisol and lactate (separately) as independent variables. Because all yellow perch scored at either 0 or 0.25 (see Results), we could not run ordinal regression for yellow perch and instead ran a binary logistic regression, again with RAMP score as the dependent variable and cortisol and lactate as independent variables. Analyses were performed using R version 3.2.1 (R Core Team, Vienna, Austria), with significance assessed at *P* < 0.05.

## Results

**Figure 2:**
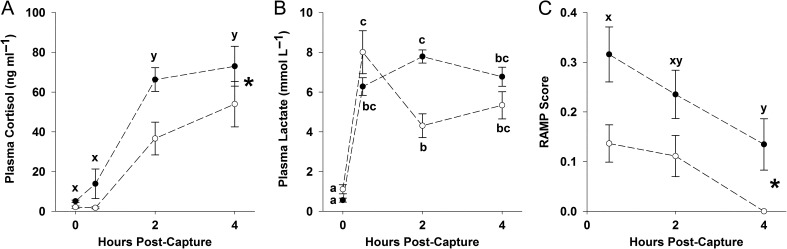
(**A**) Cortisol concentration, (**B**) lactate concentration and (**C**) RAMP score for both bluegill (black circles) and yellow perch (open circles) sampled at 0, 0.5, 2 or 4 h after ice-angling capture. The asterisk (*) on panel A indicates a significant effect of species for plasma cortisol concentration as determined by two-way analysis of variance (ANOVA), and the asterisk on panel C indicates a significant effect of species in driving whether or not fish had a non-zero RAMP score as determined by binary logistic regression. For both (A and C), significant letters (xy) indicate differences between holding time treatments. For plasma lactate (B) a significant species × holding time interaction was found, as such significant differences between individual species × holding time blocks are indicated by letters (abc).

Significant effects of both species and holding time were found for plasma cortisol (Table 2). Cortisol values were not significantly elevated above baselines at 0.5 h for either species, but by 2 h were significantly higher than baselines (Fig. 2A). Cortisol levels were not significantly different between 2 and 4 h for either species but remained significantly elevated above baseline levels (Fig. 2A). Across all holding times, cortisol concentrations in bluegill were 58% higher than yellow perch (Fig. 2A).

**Table 2:** Statistical Output for two-way analysis of variance tests (ANOVA) assessing the effect of species (bluegill, yellow perch), and holding time (baseline, 30 min, 2 h, 4 h), and their interaction on concentrations of plasma lactate and cortisol. Significant results are given in bold

	Plasma cortisol	Plasma lactate
Species	***F* = 8.19; DF = 1,89; *P* = 0.002**	*F* = 4.13; DF = 1,92; *P* = 0.07
Holding time	***F* = 33.77; DF = 3,89; *P* < 0.001**	***F* = 67.71; DF = 3,92; *P* < 0.001**
Species × holding time	*F* = 1.04; DF = 3,89; *P* = 0.37	***F* = 9.34; DF = 3,92; *P* < 0.001**

In the case of plasma lactate, a significant species × holding time interaction was detected (Table 2). For both species, lactate levels were significantly elevated relative to baseline values by 0.5 h post-capture (Fig. 2B). For bluegill, lactate levels remained elevated after 0.5 h, but for yellow perch, lactate levels declined by 54% from 0.5 to 2 h post-capture, but remained significantly higher than baseline levels (Fig. 2B).

Both species and holding time had significant effects on RAMP scores (Table 3). On average, RAMP scores were 2.7 times higher for bluegill at each holding time compared to yellow perch, indicating greater reflex impairment (Fig. 2C). RAMP scores across species declined by 70% from 0.5 to 4 h (Fig. 2C). Body flex was the reflex most often impaired for both species (30 out of 48 bluegill, 10 out of 28 yellow perch) and was the only RAMP metric observed to be impaired in yellow perch. As for the other reflex metrics, 10 out of 48 bluegill showed an impaired tail grab reflex and 6 out of 48 bluegill showed an impaired orientation response. These reflex-specific differences explain why RAMP scores were consistently lower for yellow perch than for bluegill. Vestibular-ocular response was not impaired in any of the fish in this study.

**Table 3:** Effect sizes taken from binary logistic regression model assessing the effect of species, holding time and fish length on whether or not a fish showed impairment for any of the four RAMP metrics assessed. Two-way interactions were non-significant and were removed from the model. The effect of the intercept (constant) is also included, statistically significant factors are given in bold

	*B*	S.E.	Wald	df	*P*
**Species**	**1.28**	**0.56**	**5.23**	**1**	**0.02**
**Holding time**	−**0.01**	**0.003**	**9.44**	**1**	**0.002**
Fish length (mm)	0.01	0.01	0.95	1	0.32
Constant	−1.33	1.95	0.47	1	0.49

**Figure 3:**
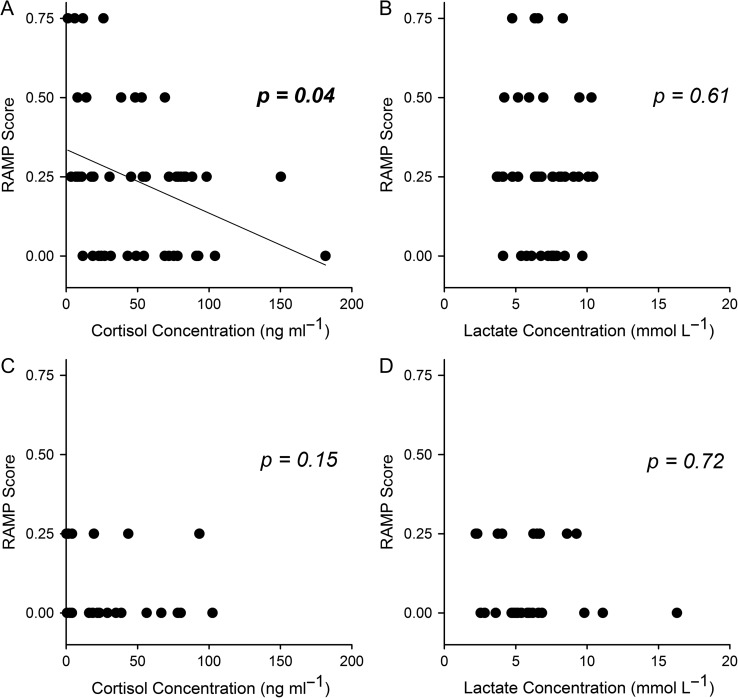
Relationships between (**A**) RAMP score and bluegill cortisol concentration, (**B**) RAMP score and bluegill lactate concentration, (**C**) RAMP score and yellow perch cortisol concentration and (**D**) RAMP score and yellow perch lactate concentration. Dots represent individual fish. *P*-values for bluegill on panels A and B derived from an ordinal regression with RAMP score as the dependent variable and plasma metric concentrations as independent variables, the regression line in panel A indicates a significant negative relationship between cortisol concentration and RAMP score for bluegill. For yellow perch, only two RAMP scores were recorded (0 or 0.25), so *P*-values are derived from a binary logistic regression model with RAMP score as the dependent variable and plasma metric concentrations as independent variables.

A significant negative relationship was found between cortisol concentrations and RAMP score for bluegill (Fig. 3A, *t* = −2.05, *P* = 0.04), but not for yellow perch (Fig. 3C, *P* = 0.15). Lactate was not related to RAMP score for either perch or bluegill (*P* > 0.61 for both, Fig. 3)

## Discussion

The process of ice-angling induces stress in captured fish, as evidenced by significant rises in plasma cortisol levels. Cortisol, the primary stress hormone in fish, is released in response to acute stressors for the purpose of activating energy stores and mediating the exchange of ions across gill membranes (Bonga, 1997; Gesto et al., 2014). While this response was indeed activated in ice-angled fish, the magnitude of the stress response observed was far lower than that seen in previous studies of bluegill and yellow perch. For instance, peak cortisol values measured 45 min after a 3 min air exposure have ranged from 177 ng ml^−1^ to upwards of 500 ng ml^−1^, respectively (Cook et al., 2012; Cousineau et al., 2014). In yellow perch, peak cortisol concentrations have been shown to vary between 107 and 170 ng ml^−1^ across a range of temperatures (Eissa and Wang, 2013). Two studies of the closely related Eurasian Perch *Perca fluviatilis* reported peak cortisol levels of ~200 ng ml^−1^ (Jentoft et al., 2005) and ~120 ng ml^−1^ measured 0.5 h after a 1 min air exposure stressor (Acerete et al., 2004). In the present study, the highest cortisol levels only reached ~53 and ~73 ng ml^−1^ 4 h post-capture for yellow perch and bluegill, respectively, well below peak levels reported in prior studies. This result is in concordance, however, with previous work on northern pike, which also showed a lower cortisol response following ice capture compared to capture in warm conditions (Louison et al., 2017). It should be noted that in both the present study and the previous study of northern pike water depths were relatively low (<2 m), which could have reduced the amount of stress on the captured fish, as capture at greater depth has been found to be linked to higher stress levels and risk of mortality (Campbell et al., 2010; Schramm et al., 2010). Additionally, because we did not assess cortisol levels more than 4 h after capture, this study does not address whether cortisol levels in ice-angled fish continue to rise after 4 h and could reach levels close to those seen in previous work. Nonetheless, it appears that ice-angling capture and handling in yellow perch and bluegill induces a less severe physiological stress response than capture and release at warmer temperatures, albeit one that may be relatively delayed and prolonged.

Plasma cortisol levels did not rise over baseline levels until 2 h post-capture, and were still elevated at 4 h. This differs from studies conducted at warmer temperatures, which have generally described cortisol levels reaching a peak within 1 h of the onset of a stressor (Galloway and Kieffer, 2003; Meka and McCormick, 2005; Vanlandeghem et al., 2010) and returning to near baseline levels by 4 h (Hyvarinen et al., 2004; Gesto et al., 2013; Jutfelt et al., 2013). However, this delayed recovery curve is similar to that seen in northern pike following ice-angling (Louison et al., 2017), as well as in other studies examining recovery at low temperatures. In one such example, cortisol levels in turbot *Scophthalmus maximus* held at 10°C did not reach their peak until 2 h post-exercise (Van Ham et al., 2003). In another example, peak cortisol levels in hybrid striped bass *Morone chrysops* × *Morone saxatilis* held at 5°C were not reached until 6 h following confinement stress (Davis, 2004). In both of these cases, peak cortisol levels were also much lower in fish stressed at low compared to high temperatures. One potential explanation for this is that reduced temperatures may be inhibiting enzyme and receptor-binding activity in the hypothalamus-pituitary-interrenal (HPI) axis, which is activated in response to stress (Bonga, 1997). The HPI axis produces cortisol as a result of an initial production of corticotropin releasing factor in the hypothalamus, which then stimulates the production of adrenocorticotropic hormone (ACTH) in the pituitary. ACTH is ultimately transported via the blood to the interrenal cells of the head kidney where cortisol is produced, and reduced temperatures could inhibit enzyme and receptor-binding activity at any of these stages. This inhibition of the stress pathway at cold temperatures would, in turn, lead to both a reduction in the magnitude of the response and a delay in the initial increase in plasma cortisol levels following a stressor (Barton and Schreck, 1987; Lankford et al., 2003). Additionally, reduced clearance of cortisol during the recovery phase could also be linked to lower metabolic rates and enzymatic activity (Van Ham et al., 2003; Davis, 2004), as well as a reduction in diffusion rates of cortisol out of the fish’s body through the gill membranes at low temperatures (Pottinger and Yeomans, 1999). Regardless of the mechanism, it appears that yellow perch and bluegill angled through the ice in winter do not begin to exhibit a stress response until >30 min after experiencing the stressor, unlike the typical response seen under warmer conditions.

Plasma lactate was elevated following ice-angling capture and unlike cortisol rose above baseline levels by 0.5 h. Significantly elevated lactate observed 0.5 h following capture indicates that anaerobic activity is still occurring at these cold temperatures (Brett, 1964), as lactate is produced in response to intense exercise and/or oxygen deprivation, as the organism shifts from anaerobic to aerobic metabolism (Wood, 1991). While lactate concentrations rose quickly, the magnitude of the response appears dampened compared to previous studies. In this study, the peak lactate concentrations for bluegill (7.7 mmol l^−1^) and yellow perch (8.0 mmol l^−1^) post-stress were lower than reported in studies of the response to in summer, for instance in peacock bass *Cichla ocellaris* (Bower et al., 2016), bonefish *Albula* spp. (Brownscombe et al., 2015), and largemouth bass (Brownscombe et al., 2014). The reduced lactate production observed in this study compared to this prior work may have resulted from reduced enzymatic activity as a result of low temperatures, specifically reduced lactate dehydrogenase activity (Wieser et al., 1986). Alternatively, it could be unrelated to temperature and related instead to the short times needed to capture fish, as longer angling durations require longer periods of anaerobic exercise and lead to greater lactate production (Brownscombe et al., 2015). Finally, the differences seen between this study and prior work could simply reflect basic differences in lactate production seen among species, regardless of temperature or angling method (Pottinger, 2010). On this score, it should be noted that peak lactate concentrations in yellow perch and bluegill were lower than the ~14 ng mmol l^−1^ seen previously in ice-angled northern pike (Louison et al., 2017), demonstrating that lactate production following capture in winter differs by species, even though peak values seen in yellow perch and bluegill were similar. Overall ice-angling capture leads to relatively low production of lactate in bluegill and yellow perch, and, unlike for cortisol, rises in lactate following capture are not delayed.

In contrast to plasma stress metrics, RAMP scores indicated that maximum impairment was present in fish shortly after capture before showing signs of recovery at succeeding time points. This pattern was identical for bluegill and yellow perch, although RAMP scores were higher in bluegill than in yellow perch throughout the recovery period. Rather than being positively correlated with plasma metrics, RAMP scores in ice-angled bluegill (but not yellow perch) were significantly and negatively associated with cortisol levels, while no relationship was found between RAMP and lactate for either species. It is presumed that reflex impairment has a basis in physiological pathways (Davis, 2010) that also drive differences in other measurements of stress (lactate, cortisol, glucose, ions, etc.), but concordance between reflex impairment and blood plasma measures have been inconsistent. Blood plasma stress indicators were not associated with RAMP scores in Coho salmon *Oncorhynchus kisutch* (Raby et al., 2012) or in bonefish (Brownscombe et al., 2015), while reflex impairment and plasma lactate were correlated in snapper *Pagrus auratus* Forster (McArley and Herbert, 2014). High RAMP scores observed shortly after capture by ice angling indicate that an individual fish is out of homoeostasis; therefore, low cortisol concentrations and high RAMP scores observed in fish sampled at 0.5 h likely indicate that individual fish had yet to respond physiologically to restore homoeostasis. The purpose of cortisol of is to restore homoeostasis following a stressor (Bonga, 1997), and if cortisol production is delayed due to low temperatures it is possible that the result could be impaired vitality due to an inability to restore homoeostasis shortly after capture. Regardless of whether cortisol concentrations and RAMP are mechanistically connected or not, the present study shows at the least that the use of RAMP may provide insights into fish recovery under winter conditions that analysis of plasma metrics alone may miss. While some prior work has shown RAMP to be more effective at predicting mortality than analysis of plasma metrics (Raby et al., 2012), future work will be needed to assess which assessment (plasma metrics or RAMP) is a better indicator of mortality risk in ice-angled sportfish species.

Bluegill and yellow perch showed significant differences in all aspects of the response to angling, as shown by plasma indicators and reflex responsiveness. While the magnitude of the rise in lactate levels was low in both yellow perch and bluegill, the recovery trajectory of lactate was different among species as shown by a significant species × holding time interaction. For plasma cortisol, the recovery trajectory (or lack thereof) was similar between species, however, cortisol levels were significantly higher across holding times in bluegill than in yellow perch. Between-species differences in plasma indicators carried over to differences in reflex responsiveness, as bluegill showed greater levels of reflex impairment across holding times as compared to yellow perch. While fish size can influence the magnitude of the stress response (Meka and McCormick, 2005) and recovery (Gingerich and Suski, 2012), fish sampled in this study represented a relatively narrow size range, limiting our ability to draw firm conclusions regarding the lack of a size effect on the stress response of bluegill or yellow perch captured via ice angling in this study. Size has been found to not influence cortisol or lactate levels in ice-angled northern pike, though again the range of fish sizes examined was narrow (Louison et al., 2017). The fact that bluegill and yellow perch differed in their response is not unexpected given previous work that has shown that fish in different taxonomic groups differ in their response to stress and in their metabolic capacity (Kieffer, 2000; Pottinger, 2010; King et al., 2016). The differences in the response to ice-angling between yellow perch and bluegill could reflect adaptive differences; optimal thermal ranges for growth are slightly lower in yellow perch (16–25°C) than in bluegill (22–30°C) (McDermot and Rose, 2000), which could lead to bluegill being more sensitive to stress at lower temperatures (Abbink et al., 2012). However, a comparison between the maximum stress response seen in previous studies of bluegill (Cook et al., 2012; Cousineau et al., 2014) and both yellow perch (Eissa and Wang, 2013) and Eurasian perch (Acerete et al., 2004; Jutfelt et al., 2013) described earlier suggests that bluegill may simply show greater responsiveness to stress, regardless of temperature. If this is the case, bluegill may be at greater risk of post-release mortality as a result of both physiological disturbance and increased risk of post-release predation (Raby et al., 2014). However, because we did not directly assess mortality, this possibility remains largely speculative.

## Conclusions

The results of this study provide a number of insights into the response of fish to stress at cold temperatures. Cold conditions appear to dampen the stress response as shown by lower levels of cortisol and lactate, however, the angling methods utilized in this study (i.e. short fight times and the capture of fish from relatively shallow depths) may have played a role as well. Recovery of plasma metrics back to baseline is notably delayed, likely due to reduced enzymatic activity at lower temperatures. The two species that were examined showed differences in their response to ice-angling stress, this applied to both measures of plasma metrics and measures of reflex responsiveness. However, the recovery course for these two metrics was very different, as cortisol and lactate levels remained elevated throughout the time course while reflex impairment was highest shortly after capture before recovering. This difference underscores the utility in applying multiple approaches to assessing stress in fish. Examining cortisol levels exclusively, for instance, would give the researcher the impression that stress was ongoing in the fish all the way through 4 h, however, the use of RAMP exclusively would lead to the conclusion that recovery was well underway. While the use of RAMP provides obvious logistical advantages given the lack of expertise and laboratory resources needed to assess it, whether it actually is superior under winter conditions in predicting eventual outcomes for captured fish cannot be determined from this study. Given the dearth of information currently available on delayed mortality following ice-angling in most targeted species, this will certainly be an area where future research can provide valuable insights by determining not only mortality rates but also how different stress indicators such as RAMP or plasma metrics relate to the likelihood of mortality.
